# Intravenous versus oral tranexamic acid in shoulder arthroplasty

**DOI:** 10.1007/s00402-026-06426-w

**Published:** 2026-07-19

**Authors:** Tarishi Parmar, Akin Adio, Mohammad Daher, Adam Khan, John Horneff, Joseph Abboud

**Affiliations:** 1 Sidney Kimmel Medical College, Thomas Jefferson University, Philadelphia, USA; 2 Division of Shoulder and Elbow Surgery, Rothman Orthopaedics, Philadelphia, USA; 3 Perelman School of Medicine, University of Pennsylvania, Philadelphia, USA; 4 Division of Shoulder and Elbow Surgery, Rothman Orthopaedics, Philadelphia, USA; 5 Southern California Permanente Medical Group, Kaiser Permanente, Panaroma city, USA; 6 Division of Shoulder and Elbow Surgery, Department of Orthopaedics, University of Pennsylvania, Philadelphia, USA

**Keywords:** Shoulder arthroplasty, Tranexamic acid, Blood loss, Transfusion, Oral vs intravenous, Cost-effectiveness

## Abstract

**Purpose:**

Tranexamic acid (TXA) is used in shoulder arthroplasty to reduce blood loss. While intravenous (IV) TXA is standard, oral TXA offers advantages in cost and administration. This study compares 90-day complications and postoperative laboratory values between IV and oral TXA in total shoulder arthroplasty (TSA).

**Methods:**

A retrospective cohort study using TriNetX identified adults undergoing primary TSA (2005–2025) receiving TXA on the day of surgery, stratified by route (IV vs. oral). One-to-one propensity score matching was performed. Ninety-day outcomes included transfusion, thromboembolic events, cardiac and renal complications, neurologic events, infection, wound dehiscence, readmissions, emergency department visits, opioid utilization, and mortality. Postoperative laboratory outcomes included hemoglobin, erythrocyte count, hematocrit, and platelet count. Outcomes were compared using odds ratios (ORs) with 95% confidence intervals (CIs) and t tests where appropriate.

**Results:**

After matching, 5722 patients were included in each cohort. No significant differences were observed between IV and oral TXA in 90-day transfusion rates (*P* = 0.873) or thromboembolic events, including deep vein thrombosis and pulmonary embolism. Rates of myocardial infarction, stroke, cardiac ischemia, acute renal failure, infection, readmission, emergency department visits, opioid use, and mortality were also comparable between groups (all *P* > 0.05). Postoperative laboratory values, including hemoglobin, erythrocyte count, hematocrit, and platelet count, did not differ significantly between IV and oral TXA cohorts (all *P* > 0.14).

**Conclusion:**

In this large propensity-matched analysis, oral TXA demonstrated equivalent safety, complication rates, and postoperative laboratory profiles compared with IV TXA following TSA. These findings support oral TXA as a clinically effective alternative to IV administration, offering potential advantages in cost reduction and resource utilization without compromising patient safety. Future prospective studies should evaluate optimal dosing strategies, patient-specific selection criteria, and formal cost-effectiveness analyses to further define the role of oral TXA in shoulder arthroplasty pathways.

**Level of evidence:**

III, Retrospective Cohort Study.

**Supplementary Information:**

The online version contains supplementary material available at 10.1007/s00402-026-06426-w.

## Introduction

Total shoulder arthroplasty is being performed with increasing frequency as indications expand and the population ages [4]. Despite advances in surgical technique and perioperative care, clinically significant blood loss remains common during shoulder arthroplasty [3]. Reported intraoperative blood loss often ranges from several hundred milliliters to more than one liter, depending on implant type and procedural complexity [10]. Prior studies have reported postoperative transfusion rates ranging from 7.4 to 43% following shoulder arthroplasty, a substantial risk that may translate into higher rates of postoperative infection, longer hospital length of stay, increased healthcare costs, and greater mortality [6]. Collectively, these findings highlight perioperative blood loss as an ongoing and important clinical challenge in shoulder arthroplasty.

Tranexamic acid has become an established blood-sparing agent in shoulder arthroplasty and is widely used to reduce perioperative bleeding and transfusion requirements [18]. Most contemporary protocols rely on intravenous (IV) tranexamic acid because of its rapid onset, predictable bioavailability, and familiarity among anesthesia and surgical teams [26]. As a result, IV administration has emerged as the predominant route in both clinical practice and the existing shoulder arthroplasty literature [1]. However, persistent concerns remain regarding IV tranexamic acid, including potential thromboembolic risk, medication cost, dosing complexity, and perioperative logistical burden [17]. Oral tranexamic acid has therefore emerged as a potentially safer, simpler, and lower-cost alternative, with established efficacy in other arthroplasty settings [7]. Despite this, data directly comparing the effectiveness and safety of oral versus IV tranexamic acid in shoulder arthroplasty remain limited, leaving an important gap in the literature.

This study aims to evaluate the association between tranexamic acid administration route and postoperative outcomes in patients undergoing shoulder arthroplasty using a large, real-world, multicenter cohort. We define exposure based on receipt of oral or IV tranexamic acid in the perioperative period. Using 1:1 propensity score matching across demographic, clinical, and procedural variables, we compare short-term postoperative outcomes between patients receiving oral versus IV tranexamic acid. We hypothesize that oral tranexamic acid will demonstrate comparable effectiveness and safety to IV tranexamic acid, supporting its use as a simplified and lower-cost alternative in shoulder arthroplasty.

## Methods

### Data source

Data for this study were derived from the TriNetX Research Network, a federated, global electronic health record database containing information on over 195 million patients from participating healthcare systems. The platform enables access to de-identified, longitudinal clinical data, including diagnostic codes, procedural information, medication records, and laboratory results. Data contributed by participating institutions are standardized using TriNetX-supported syntactic and semantic mapping to a unified common data model. Each patient is assigned a secure, encrypted identifier, permitting longitudinal follow-up across encounters while maintaining patient confidentiality. As all data are de-identified and analyzed in aggregate, this study was exempt from institutional review board approval.

### Cohort selection

A retrospective cohort study was conducted using the TriNetX Research Network. Adult patients who underwent primary total shoulder arthroplasty (TSA) between 2005 and 2025 were identified using relevant procedural codes, with the database queried on January 5, 2026. Patients were included if they received tranexamic acid (TXA) on the day of surgery and were stratified based on route of administration: IV versus oral. To isolate primary elective cases, patients were excluded if they underwent revision shoulder arthroplasty, sustained acute traumatic injuries, or received TXA outside the perioperative window. Patients without a minimum of 90 days of postoperative follow-up were excluded from the analysis.

### Study population and propensity matching

The primary analysis compared postoperative outcomes between patients who received IV TXA and those administered oral TXA during the perioperative period. To minimize baseline differences between groups, propensity scores were generated using multivariable logistic regression that included demographic factors and relevant clinical comorbidities (Table 1). Cohorts were then matched 1:1 using a greedy nearest-neighbor algorithm with a caliper of 0.1 standard deviations of the pooled propensity score. Covariate balance was reassessed after matching to confirm adequate comparability before outcome analyses were performed.

**Table 1:** Characteristics of cohorts before and after propensity score matching

Characteristics	Cohort	Before matching			After matching		
		Patients	% Patients	*P* value	Patients	% Patients	*P* value
*Demographics*							
Age at Index	IV Oral	45537 (69.6 ± 8.95) 5723 (69.6 ± 8.81)	100% 100%	0.699	5722 (69.5 ± 8.83) 5722 (69.6 ± 8.81)	100% 100%	0.334
White	IV Oral	40,153 5182	88.18% 90.55%	< 0.001	5145 5182	89.92% 90.56%	0.244
Female	IV Oral	25,256 3157	55.46% 55.16%	0.668	3178 3156	55.54% 55.16%	0.679
Not Hispanic or Latino	IV Oral	37,579 5374	82.52% 93.90%	< 0.001	5350 5373	93.50% 93.90%	0.376
Hispanic or Latino	IV Oral	1417 130	3.11% 2.27%	< 0.001	150 130	2.62% 2.27%	0.226
Black or African American	IV Oral	3071 323	6.74% 5.64%	0.002	333 323	5.82% 5.64%	0.687
Male	IV Oral	20,235 2566	44.44% 44.84%	0.565	2541 2566	44.40% 44.84%	0.638
Asian	IV Oral	553 21	1.21% 0.37%	< 0.001	23 21	0.40% 0.37%	0.763
*Diagnosis*							
Diabetes mellitus	IV Oral	11,324 1480	24.87% 25.86%	0.102	1426 1479	24.92% 25.85%	0.255
Acute Kidney Failure and Chronic Kidney Disease	IV Oral	7658 923	16.82% 16.13%	0.188	867 923	15.15% 16.13%	0.150
Heart failure	IV Oral	4425 553	9.72% 9.66%	0.895	501 553	8.76% 9.66%	0.092
Tobacco Use	IV Oral	2222 331	4.88% 5.78%	0.003	314 331	5.49% 5.78%	0.491
Hypertensive diseases	IV Oral	30,572 3,910	67.14% 68.32%	0.072	3859 3909	67.44% 68.31%	0.317
Other diseases of liver	IV Oral	4514 494	9.91% 8.63%	0.002	487 494	8.51% 8.63%	0.815
Atrial fibrillation and flutter	IV Oral	4967 682	10.91% 11.92%	0.022	659 682	11.52% 11.92%	0.504
Long term (current) use of anticoagulants	IV Oral	4728 647	10.38% 11.30%	0.032	594 646	10.38% 11.29%	0.118
Other venous embolism and thrombosis	IV Oral	2294 279	5.04% 4.87%	0.595	248 279	4.33% 4.88%	0.167
Pulmonary embolism	IV Oral	1144 138	2.51% 2.41%	0.645	133 138	2.32% 2.41%	0.758
Alcohol related disorders	IV Oral	2723 337	5.98% 5.89%	0.783	375 337	6.55% 5.89%	0.141
Opioid related disorders	IV Oral	1391 159	3.05% 2.78%	0.250	165 159	2.88% 2.78%	0.735
Ischemic heart diseases	IV Oral	10,604 1405	23.28% 24.55%	0.033	1282 1404	22.40% 24.54%	0.007
Cerebral infraction	IV Oral	2084 270	4.58% 4.72%	0.630	239 270	4.18% 4.72%	0.160
Transient cerebral ischemic attacks and related syndromes	IV Oral	47,318 98,740	43.67% 15.00%	< 0.001	44,952 45,052	42.47% 42.56%	0.198
Epilepsy and recurrent seizures	IV Oral	1014 134	2.23% 2.34%	0.581	114 134	1.99% 2.34%	0.199
Overweight and obesity	IV Oral	15,378 2062	33.77% 36.03%	< 0.001	2020 2061	35.30% 36.02%	0.424
*Laboratory*							
BMI	IV Oral	38,479 (30.6 ± 6.55) 5122 (30.7 ± 6.48)	84.50% 89.50%	0.188	5199 (30.5 ± 6.36) 5121 (30.7 ± 6.48)	90.86% 89.50%	0.054
Hematocrit [volume fraction] of blood	IV Oral	41,436 (39.8 ± 5.82) 5377 (40.1 ± 4.98)	90.99% 93.95%	0.001	5354 (40.2 ± 4.85) 5377 (40.1 ± 4.98)	93.55% 93.95%	0.375
Hemoglobin [mass/volume] in blood	IV Oral	41,214 (13.3 ± 1.76) 5372 (13.3 ± 1.73)	90.51% 93.87%	0.007	5338 (13.3 ± 1.7) 5372 (13.3 ± 1.73)	93.27% 93.87%	0.697
Erythrocytes [#/volume] in blood	IV Oral	44,757 (4.2 ± 1.17) 4784 (4.4 ± 0.59)	90.03% 92.98%	< 0.001	4782 (4.39 ± 0.67) 4784 (4.4 ± 0.59)	92.94% 92.98%	0.499
Platelets [#/volume] in blood	IV Oral	40,868 (249 ± 73.5) 5362 (247 ± 74.8)	89.75% 93.69%	0.234	5323 (247 ± 73.1) 5362 (247 ± 74.8)	93.01% 93.69%	0.859

### Outcomes

The primary outcomes were 90-day postoperative medical and surgical complications following total shoulder arthroplasty. These included transfusion, deep vein thrombosis, pulmonary embolism, myocardial infarction, seizures, visual disturbances, ischemic stroke or transient ischemic attack, cardiac ischemia, acute renal failure, opioid use, periprosthetic joint infection, readmission, emergency department visits, surgical site infection, wound dehiscence, and all-cause mortality.

Secondary outcomes included postoperative laboratory values obtained the day after surgery, including hemoglobin, erythrocyte count, hematocrit, and platelet count. Outcomes with fewer than ten events were reported as “≤10” in accordance with TriNetX privacy policies. All outcomes were assessed within their predefined postoperative time windows.

### Statistical analysis

Statistical analyses were conducted within the TriNetX Analytics environment, which employs Java-, R-, and Python-based computational frameworks. Group comparisons for categorical postoperative outcomes were reported as odds ratios with corresponding 95% confidence intervals, with chi-square or Fisher’s exact tests used as appropriate based on event frequency. A two-tailed P value < 0.05 was considered statistically significant. All analyses were performed on propensity score–matched cohorts to maintain balance in baseline characteristics.

## Results

### Overall cohort demographics

Before matching, the IV TXA cohort included 45,537 patients and the oral TXA cohort included 5,723 patients. After 1:1 propensity score matching, 5,722 patients remained in each group. Following matching, baseline demographic characteristics were well balanced between cohorts. Mean age at surgery was similar between the IV and oral TXA groups (69.5 ± 8.83 vs. 69.6 ± 8.81 years, *P* = 0.33), as was sex distribution (female: 55.5% vs. 55.2%, *P* = 0.68). Racial and ethnic composition was comparable, including proportions identifying as White (89.9% vs. 90.6%, *P* = 0.24) and Not Hispanic or Latino (93.5% vs. 93.9%, *P* = 0.38).

Comorbidity profiles were also well matched, with no statistically significant differences observed across major medical conditions, including hypertension, diabetes mellitus, ischemic heart disease, atrial fibrillation, renal disease, heart failure, thromboembolic disease, seizure disorders, tobacco use, alcohol-related disorders, or opioid-related disorders. Long-term anticoagulant use was also balanced between cohorts. Baseline laboratory values were similarly balanced following matching, including hemoglobin (13.3 ± 1.7 vs. 13.3 ± 1.73 g/dL, *P* = 0.697), hematocrit (40.2 ± 4.85% vs. 40.1 ± 4.98%, *P* = 0.375), erythrocyte count (4.39 ± 0.67 vs. 4.40 ± 0.59 × 10⁶/µL, *P* = 0.499), and platelet count (247 ± 73.1 vs. 247 ± 74.8 × 10³/µL, *P* = 0.859). Body mass index was also comparable between cohorts (30.5 ± 6.2 vs. 30.7 ± 6.5 kg/m²), indicating adequate balance across all matched variables.

### Ninety-day outcomes and postoperative laboratory results

At 90 days following total shoulder arthroplasty, no significant differences in postoperative complications were observed between patients receiving IV and oral tranexamic acid (Table 2). Rates of transfusion (1.53% vs. 1.49%, OR 1.03, *P* = 0.873), deep vein thrombosis (1.49% vs. 1.49%, OR 1.00, *P* = 1.000), pulmonary embolism (0.83% vs. 0.91%, OR 0.92, *P* = 0.675), myocardial infarction (0.45% vs. 0.62%, OR 0.73, *P* = 0.232), seizures (2.08% vs. 2.27%, OR 0.92, *P* = 0.505), and visual disturbances (0.51% vs. 0.34%, OR 1.50, *P* = 0.179) were comparable between cohorts. Similarly, there were no significant differences in ischemic stroke/transient ischemic attack, cardiac ischemia, acute renal failure, periprosthetic joint infection, readmission, emergency department visits, surgical site infection, wound dehiscence, opioid use, or all-cause mortality.

**Table 2:** Ninety-day outcomes after total shoulder arthroplasty with intravenous versus oral TXA

Outcome	Incidence (%)		Odds ratio	95% confidence interval	*P* value
	IV	Oral			
*90-days*					
Transfusion	1.53%	1.49%	1.03	(0.751,1.402)	0.873
DVT	1.49%	1.49%	1.00	(0.73,1.369)	1.000
PE	0.83%	0.91%	0.92	(0.607,1.381)	0.675
MI	0.45%	0.62%	0.73	(0.429,1.23)	0.232
Seizures	2.08%	2.27%	0.92	(0.704,1.188)	0.505
Visual changes	0.51%	0.34%	1.50	(0.827,2.731)	0.179
Ischemic stroke/TIA	1.21%	1.63%	0.74	(0.535,1.026)	0.070
Cardiac ischemia	15.03%	16.10%	0.92	(0.829,1.023)	0.126
Acute renal failure	2.40%	2.67%	0.90	(0.705,1.145)	0.386
Opioids	99.83%	99.85%	0.89	(0.343,2.305)	0.808
PJI	1.27%	1.49%	0.85	(0.61,1.174)	0.317
Readmission	14.52%	13.72%	1.07	(0.957,1.191)	0.241
ED visits	8.62%	8.79%	0.98	(0.855,1.121)	0.756
Mortality	0.30%	0.23%	1.33	(0.631,2.823)	0.449
SSI	0.76%	0.74%	1.03	(0.659,1.597)	0.910
Wound dehiscence	0.35%	0.47%	0.74	(0.414,1.321)	0.306

*DVT* deep vein thrombosis, *PE* pulmonary embolism, *MI* myocardial infraction, *TIA* transient ischemic attack, *PJI* peri-prosthetic joint infection, *ED* emergency department, *SSI* surgical site infection

Postoperative laboratory values obtained the day after surgery were comparable between groups as well (Table 3). Mean hemoglobin levels were similar in the IV and oral TXA cohorts (11.44 vs. 11.40 g/dL, *P* = 0.339), as were erythrocyte counts (3.75 vs. 3.77 × 10⁶/µL, *P* = 0.145), hematocrit (34.54% vs. 34.42%, *P* = 0.296), and platelet counts (215.2 vs. 213.9 × 10³/µL, *P* = 0.453).

**Table 3:** Post-operative lab values after total shoulder arthroplasty with intravenous versus oral TXA

IV vs. oral	Incidence		Mean		*P* value
	IV (5722)	Oral (5722)	IV	Oral	
Hematocrit [volume fraction] of blood	3018	3511	11.43	11.40	0.339
Hemoglobin [mass/volume] in blood	2665	3091	3.75	3.77	0.145
Erythrocytes [#/volume] in blood	3067	3635	34.54	34.42	0.296
Platelets [#/volume] in blood	2662	3091	215.20	213.91	0.453

## Discussion

In this large database analysis of patients undergoing TSA administered TXA perioperatively, oral and IV tranexamic acid demonstrate comparable efficacy and safety profiles following TSA. Across more than 5,000 matched patients, the route of TXA administration did not influence transfusion requirements, perioperative hematologic indices, thromboembolic or neurologic complications, readmission, or mortality, suggesting that oral TXA is non-inferior to IV TXA in contemporary clinical practice.

Effective blood conservation remains a central objective of TXA use in arthroplasty, as perioperative blood loss and allogeneic transfusion have been consistently associated with increased postoperative morbidity, infectious complications, and healthcare utilization [5, 12, 21]. In the present study, transfusion rates were nearly identical between IV and oral TXA cohorts. Postoperative hemoglobin, erythrocyte count, and hematocrit values were similarly preserved, suggesting comparable control of perioperative blood loss between routes of administration. Evidence from lower-extremity arthroplasty provides important context for these findings. In a randomized non-inferiority trial of patients undergoing primary total hip and knee arthroplasty, DeFrancesco et al. demonstrated that oral TXA was non-inferior to IV TXA with respect to blood loss and transfusion requirements [7]. Similarly, multiple systematic reviews and meta-analyses in total hip and knee arthroplasty have reported comparable reductions in perioperative blood loss and transfusion rates between oral and IV TXA [8, 20, 26]. The findings of the present shoulder arthroplasty cohort closely parallel this established hip and knee literature, extending evidence to a population in which direct comparative data have been limited. Beyond transfusion, prior studies have demonstrated that greater perioperative blood loss and exposure to allogeneic transfusion are associated with increased short-term mortality following major surgical procedures, including joint arthroplasty [5, 12, 27]. In this context, although causality cannot be inferred given the use of all-cause mortality, the observed equivalence in postoperative hematologic parameters and transfusion rates may contribute to the similar mortality risk between the two cohorts, further supporting comparable efficacy between IV and oral TXA.

Concerns regarding TXA-related complications, particularly thromboembolic and neurologic events, have historically tempered broader adoption across surgical populations, despite increasing use in arthroplasty [15, 24]. In the present analysis, the incidence of deep vein thrombosis and pulmonary embolism was identical between oral and IV TXA cohorts, with no significant differences observed in myocardial infarction, cardiac ischemia, ischemic stroke or transient ischemic attack, seizures, visual changes or acute renal failure. These findings align with prior comparative studies and large meta-analyses comparing oral and IV TXA in total hip and knee arthroplasty have reported equivalent rates of thromboembolic events and systemic complications across administration routes, supporting a comparable safety profile [7, 23, 25]. Tranexamic acid has also been associated with an increased risk of seizures, particularly in settings involving high IV doses, owing to its effects on inhibitory neurotransmission [15]. However, IV TXA use in arthroplasty has not been shown to be associated with an increased incidence of seizures [13, 22]. While prior studies have not specifically evaluated seizure risk associated with oral TXA, the present analysis adds to the existing literature by demonstrating equivalent postoperative seizure rates between oral and IV TXA. Further, no significant differences in periprosthetic joint infection, surgical site infection, or wound dehiscence were observed between intravenous and oral TXA. These findings are consistent with prior systematic reviews in arthroplasty and fracture fixation populations, which demonstrate that TXA use does not increase postoperative infection risk and may reduce wound complications through improved hemostasis and reduced hematoma formation, regardless of route of administration [2, 19]. 

Additional postoperative outcomes, including emergency department visits, readmissions, and opioid utilization, were also comparable between groups. Collectively, our findings suggest that route of TXA administration does not significantly influence thromboembolic, neurologic, cardiac, or renal complication rates, reinforcing the overall safety equivalence of oral and IV TXA.

Beyond clinical equivalence, the route of TXA administration has meaningful practical implications. Oral TXA avoids the need for infusion preparation and perioperative administration logistics, which may simplify workflows in both inpatient and outpatient settings [16, 28]. Further, studies within the hip and knee arthroplasty literature consistently demonstrate the cost advantage of oral over IV TXA [11, 16, 28]. Kayupov et al. reported the cost of a single oral TXA dose to be approximately $14, compared with $47–$108 for IV TXA, representing a 70–90% reduction in drug-related cost [11]. Given that commonly reported perioperative dosing regimens in shoulder arthroplasty include 1 g of IV TXA and 2 g of oral TXA, these differences suggest the potential for per-patient cost savings of hundreds of dollars when oral TXA is utilized in TSA as well [9]. As the annual volume of total shoulder arthroplasty continues to rise and is projected to reach at least 37,000 procedures per year by 2040, oral TXA mediated per-case savings may translate into millions of dollars in aggregate healthcare cost savings at a national level [14]. However, because TXA dosing regimens in total shoulder arthroplasty have not yet been standardized, these projections should be interpreted with caution until future studies better define optimal dosing strategies.

Thus, given comparable efficacy, safety, and short-term outcomes between oral and IV TXA, oral administration may represent a practical alternative in appropriately selected patients undergoing TSA. Its logistical simplicity and potential cost advantages further support consideration within evolving outpatient and value-based care pathways. Future prospective studies are needed to confirm these findings and to define optimal dosing strategies and patient selection.

### Limitations

Several limitations should be considered when interpreting these findings. This study utilizes data from a large federated electronic health record network, and classification of tranexamic acid exposure relied on medication and procedural coding rather than direct verification of administration details. Although patients were categorized as receiving IV or oral TXA, the dataset does not allow determination of the exact dose, frequency, or timing of administration on the day of surgery. In particular, it is not possible to ascertain whether TXA was given preoperatively, intraoperatively, or postoperatively, which may be clinically relevant given evidence that timing influences antifibrinolytic efficacy. Perioperative blood management strategies vary across participating healthcare organizations and could independently affect bleeding-related outcomes. While propensity score matching was used to balance measured baseline characteristics, unmeasured confounding related to operative complexity, surgeon experience, or intraoperative blood loss may still influence results. Outcome assessment was limited to coded postoperative events and indirect markers of bleeding risk. Laboratory data were limited, as patients discharged on the day of surgery often lacked postoperative values taken day after surgery. Same-day labs were not considered since they could not be reliably classified as preoperative or postoperative. Further, database did not permit evaluation of change from pre- to post-operative lab values. Additionally, medication exposure was treated as a single perioperative event. The analysis could not account for preoperative anticoagulant use or postoperative thromboprophylaxis regimens, which may differentially influence bleeding or thromboembolic risk. Finally, the retrospective observational design precludes causal inference.

## Conclusion

Oral and IV tranexamic acid were associated with comparable perioperative and short-term outcomes following total shoulder arthroplasty, with no significant differences in transfusion rates, postoperative hematologic parameters, thromboembolic or neurologic complications, readmissions, or mortality. These findings support equivalent efficacy and safety between routes of administration for perioperative blood conservation in TSA. Clinically, this equivalence supports consideration of oral TXA as a practical alternative to IV administration. Oral TXA offers advantages in terms of simplified perioperative logistics and reduced drug-related costs without compromising patient outcomes. Future prospective studies are warranted to validate these findings, establish standardized dosing and timing protocols specific to shoulder arthroplasty, and evaluate outcomes in higher-risk patient populations.

## Supplementary Information

Below is the link to the electronic supplementary material.

Supplementary Material 1

## Data Availability

The data that support the findings of this study were obtained from the TriNetX research network. Access to these data is subject to institutional licensing agreements and therefore the data are not publicly available. Data may be accessed by qualified researchers through TriNetX with appropriate institutional authorization.
