# Supplementary material for: Intravenous versus oral tranexamic acid in shoulder arthroplasty
Source: Arch Orthop Trauma Surg. 2026 Jul 19;146(1):263. doi: 10.1007/s00402-026-06426-w (PMC13381376; doi:10.1007/s00402-026-06426-w)
Supplement: Supplementary file 1 — Supplementary Material 1 [file 402_2026_6426_MOESM1_ESM.docx]

**Supplementary Table**

| **Codes** | |
| --- | --- |
|  |  |
| Total Shoulder Arthroplasty | 23472, 0RRJ0JZ, 0RRK0J7, 0RRK0JZ, 0RRJ00Z, 0RRK00Z |
|  |  |
| Tranexamic Acid | 10691 |
|  |  |
| **Outcomes** |  |
| **90-day** |  |
| Transfusion | 302, 36430 |
| DVT | I82 |
| PE | I26 |
| MI | I21 |
| Seizures | G40, R56 |
| Visual Changes | H43 |
| Ischemic stroke/TIA | I63, G45 |
| Cardiac Ischemia | I20-I25 |
| Acute Renal Failure | N17, N19 |
| Opioids | N02A |
| PJI | T84.5 |
| Readmission | Visit: Inpatient encounter |
| ED-visits | Visit: emergency |
| Mortality | Deceased |
| SSI | T81.4 |
| Wound Dehiscence | T81.3 |
| **Lab Outcomes** |  |
| Hemoglobin [Mass/Volume] in Blood | 718-7 |
| Erythrocytes [#/volume] in Blood | 9012 |
| Platelets [#/volume] in Blood | 9020 |
| Hematocrit [Volume Fraction] of Blood | 9013 |
